# Efbalropendekin Alfa enhances human natural killer cell cytotoxicity against tumor cell lines *in vitro*

**DOI:** 10.3389/fimmu.2024.1341804

**Published:** 2024-03-07

**Authors:** Hesham M. Shehata, Pranay Dogra, Sarah Gierke, Patrick Holder, Shomyseh Sanjabi

**Affiliations:** ^1^ Department of Translational Medicine Oncology, Genentech Inc., South San Francisco, CA, United States; ^2^ Department of Pathology, Genentech Inc., South San Francisco, CA, United States; ^3^ Department of Protein Chemistry, Genentech Inc., South San Francisco, CA, United States

**Keywords:** IL-15, immunotherapy, NK cells, cytotoxicity, cytokine

## Abstract

IL-15 has shown preclinical activity by enhancing the functional maturation of natural killer (NK) cells. Clinical evaluation of the potential anticancer activity of most cytokines, including IL-15, has been limited by low tolerability and rapid *in vivo* clearance. Efbalropendekin Alfa (XmAb24306) is a soluble IL15/IL15-receptor alpha heterodimer complex fused to a half-life extended Fc domain (IL15/IL15Rα-Fc), engineered with mutations to reduce IL-15 affinity for CD122. Reduced affinity drives lower potency, leading to prolonged pharmacodynamic response in cynomolgus monkeys. We show that *in vitro*, human NK cells treated with XmAb24306 demonstrate enhanced cytotoxicity against various tumor cell lines. XmAb24306-treated NK cells also exhibit enhanced killing of 3D colorectal cancer spheroids. Daratumumab (dara), a monoclonal antibody (mAb) that targets CD38 results in antibody-dependent cellular cytotoxicity (ADCC) of both multiple myeloma (MM) cells and NK cells. Addition of XmAb24306 increases dara-mediated NK cell ADCC against various MM cell lines *in vitro*. Because NK cells express CD38, XmAb24306 increases dara-mediated NK cell fratricide, but overall does not negatively impact the ADCC activity against a MM cell line likely due to increased NK cell activity of the surviving cells. These data show that XmAb24306 increases direct and ADCC-mediated human NK cell cytotoxicity *in vitro*.

## 1 Introduction

Cytokines that can augment T-cell and NK-cell mediated immunity may have therapeutic applications in cancer patients (1, 2). Despite the known biology of cytokines and their role in the immune system and cancer biology, only a limited number of cytokines have been approved for cancer treatment in select indications (1, 2). This includes IFNα (e.g., hairy cell leukemia and chronic myelogenous leukemia among others) and IL-2 (aldesleukin)(e.g., advanced melanoma and metastatic RCC). This is, in part, related to poor tolerability, a narrow therapeutic index, and the poor PK behavior of these cytokines (3–5). Despite the clinical benefit of IL-2 treatment, its use is limited by its toxicity profile, requiring intensive inpatient monitoring for administration. In addition, there are biological effects of IL-2 that may limit its efficacy in enhancing anti-tumor immunity, including its ability to cause activation-induced cell death (AICD) of T cells and its immunosuppressive effects via activation of regulatory T cells (Tregs) (6). IL-15 is another cytokine with therapeutic potential to promote anti-tumor immunity via its activity on NK and T-cells (6–10). IL-2 and IL-15 share the common gamma chain (CD132) and beta-receptor subunit (CD122), but they each have a unique alpha-receptor subunit. IL-15 is trans-presented by monocytes and DCs in the context of IL-15 receptor alpha (IL-15Rα; CD215) (11). Thus, when IL-15/IL-15Rα binds to CD122 and CD132 on NK and T cells, it leads to enhanced durable cellular responses, including proliferation and cytotoxicity (11). There has been considerable effort in the development of IL-15 for clinical use with multiple modifications including potency reduction or potency enhancements (11, 12). One of the key therapeutic limitations of recombinant cytokines is their short half-lives, which in the case of gamma-cytokines like IL-15, is compounded further by the rapid clearance upon engagement and internalization through CD122 (13). Therefore, there is a need to develop cytokine therapies that have improved half-life to enhance the therapeutic index.

Efbalropendekin Alfa (XmAb24306) is an IL-15/IL-15 receptor alpha complex fused to a heterodimeric Fc domain (IL15/IL15Rα-Fc) (14, 15) currently in Phase 1 clinical development (NCT04250155, NCT05646836, NCT05243342). The IL-15 domain is engineered with mutations that are designed to reduce IL-15 affinity for its receptor. Reduced affinity drives lower potency on cells that contain the IL-15 beta receptor, which translates to increased exposure and prolonged pharmacodynamic response on target cells (14, 15). The Fc domain was further modified through the addition of mutations that enhance FcRn-mediated recycling and eliminate FcγR and C1q binding, thus prolonging serum half-life (14, 15). Acceptable tolerability and prolonged half-life (~ 2.5 to 4.5 days) of XmAb24306 in cynomolgus monkeys led to increased exposure, enhanced and durable expansion of NK cells and CD8+ T cells (16).

Natural Killer (NK) cells are cytotoxic innate immune cells that play an important role in tumor immunosurveillance by recognizing and eliminating stressed or transformed cells, a key first step in initiating the cancer immunity cycle (17, 18). Once activated, one of the mechanisms by which NK cells can directly eliminate tumor targets is via exocytosis of pre-assembled cytolytic granules containing granzyme B and perforin across the immunological synapse, ultimately inducing apoptosis and death of target cells (19, 20). NK cells can also indirectly promote antitumor activity through the release of immunoregulatory cytokines such as interferon-γ (IFN-γ), tumor necrosis factor-α (TNF-α) and granulocyte–macrophage colony-stimulating factor (21–23). NK cell function is determined by the integration of signals arising from the engagement of various germline encoded activating and inhibitory receptors (24) and by their differentiation state which is categorized based on cell surface expression of CD56 and the low affinity FcγRIIIa, CD16 (25). CD56^dim^ CD16^+^ NK cells constitute the predominant NK cell subset in peripheral blood and are considered to be the most mature subset that can mediate serial killing of malignant cells without prior activation (20, 26). CD16 is a prototypical NK cell-activating receptor as its engagement is sufficient to trigger cytotoxic activity and production of pro-inflammatory cytokines and chemokines without the requirement for additional activation through other activating receptors (27, 28). Thus, CD16^+^ NK cells are specialized in mediating antibody dependent cellular cytotoxicity (ADCC) against monoclonal antibody opsonized target cells (27). During ADCC, NK cell cytolytic activity is triggered via the cross-linking of CD16 to the Fc portion of IgG therapeutic antibodies (29, 30).

Multiple myeloma (MM) is a hematologic neoplasm characterized by monoclonal expansion of malignant plasma cells in the bone marrow (BM) (31, 32). High expression of CD38 on the surface of malignant plasma cells in MM led to the development of CD38 targeting monoclonal antibodies (mAbs), daratumumab (dara) and isatuximab for the treatment of MM (33–38). ADCC has been reported to be an important mechanism of action for CD38 targeting mAbs. Primary resistance to dara has been linked to loss of CD38 expression, and there is a trend toward higher response rates with increasing CD38 levels on MM cells (39). CD38 is also expressed on other immune cells including NK cells. The use of such mAbs, including dara (40–42) can lead to ADCC-mediated fratricide of CD38+ NK cells resulting in rapid decline of NK cells in peripheral blood and bone marrow of MM patients (40).

Here, we investigated the role of XmAb24306 in enhancing the NK cell effector function and killing of various tumor cell lines. We show that incubation of primary human NK cells with XmAb24306 enhances their direct cytotoxic functionality against various tumor cell lines and 3D colorectal tumor spheroids and also increases ADCC activity against various MM cell lines. Together, these *in vitro* data demonstrate the potential of XmAb24306 as an immunotherapeutic for enhancing NK cell cytolytic function.

## 2 Materials and methods

### 2.1 Human cancer cells

Human leukemia (K562), breast (MDA-MB231), ovarian (OVCAR8), lung (NCI-H46), colon (HCT116), bladder (RT112), and multiple myeloma (RPMI 8226, NCI-H929, MM1.S and U266) cells were stored at Genentech’s internal cell line repository, gCell. K562, MDA-MB231, NCI-H460, HCT-116, RPMI 8226, NCI H929 and U266 cells were obtained from ATCC. OVCAR8 cells were obtained from NCI-Frederick Cancer DCTD. RT112 were obtained from DSMZ and MM1.S cells were obtained from Northwestern University. Tumor cells were cultured in culture medium (RPMI 1640, 10% FBS, 1% penicillin/streptomycin, 1% glutamine). For functional assays, adherent tumor cells were isolated into single cells with 0.5% trypsin/EDTA and following centrifugation, were subsequently resuspended in culture medium.

### 2.2 Peripheral blood NK cell isolation

Whole blood samples from healthy donors were supplied through the Samples for Science program, an IRB approved research program operated out of the Genentech Campus Health Center. NK cells were purified via negative selection using the RosetteSep Human NK Isolation Kit (StemCell Technologies) according to the manufacturer’s protocol. Briefly, 500 μL of reagent were added per 10 ml of whole blood for at least 30 min. Subsequently, whole blood samples were diluted 1:1 with 1X sterile PBS and overlaid on Ficoll-Paque (cat #45-001-749) and centrifuged for 20 min at 1,200 g (no brakes). The interface containing enriched NK cells was isolated and NK cells were enumerated and purity of NK cells (Live-Dead-CD45+CD3-CD14/19-CD56+) as determined by flow cytometry was >90% for all participants evaluated (**Supplementary Data 1**).

### 2.3 NK cell activation

Enriched blood NK cells were cultured in culture medium in the presence or absence of 10 µg/ml XmAb24306 for 18–20 hrs. Primed viable NK cells (>95% viability) (**Supplementary Data 1**) were then washed, enumerated and co-cultured with target cells at various E:T ratios where specified. The same number of NK cells for XmAb24306 and untreated conditions were added to target cells. The viability of untreated or XmAb24306 treated NK cells remained at 90-95% during various time points of the K562 cytotoxicity assay.

### 2.4 Cytotoxicity assay

NK cell cytotoxicity was performed using a flow cytometry cytotoxicity assay based on the measurement of the uptake of Annexin V and 7AAD dyes in target tumor cells. The target cancer cells were labeled with CellTrace Violet (0.5µM; ThermoFisher Scientific) according to the manufacturer’s instructions and then suspended in culture medium and plated at a density of 5×10^4^ cells per well in 96-well plates. Enriched NK cells (effector) that were either pre-activated with XmAb24306 (10µg/ml) overnight or cultured in control media were then added at various E:T ratios as indicated. The plates were centrifuged at 200 g for 2 min and incubated for the indicated times at 37°C, 5% CO_2_. Control wells were set up without NK cells to monitor background tumor cell death in the absence of effector NK cells. At the end of the cytotoxicity assay, the cell mixtures were stained for surface antibodies and subsequently resuspended in Annexin V binding buffer (1X) containing 1:100 dilution of Annexin V-PE and 7AAD as per the manufacturer’s protocol. Dead cells were reported as the sum of AnnexinV+7AAD double positive cells and 7AAD+AnnexinV- single positive cells. Apoptotic cells were reported as 7AAD-Annexin V+. Tumor cell and NK cell death were monitored using either a BD Fortessa or Cytek Aurora flow cytometers and analyzed using either FlowJo software (Version 10.6.2, Treestar) or cloud based OMIQ. Specific NK induced cell lysis was calculated using the following formula: % NK cell induced death = (% tumor death in tumor + NK cells) - (% tumor death in tumor - NK cells). K562 and solid tumor cell line killing assays were tested with at least 6 individual donors.

### 2.5 Antibody-dependent cell cytotoxicity

To evaluate the ADCC capacity of XmAb24306 primed NK cells, we measured NK cell mediated cytotoxicity against MM cells in the presence of the anti-CD38 monoclonal antibody dara. Briefly, target MM cell lines including RPMI 8226, NCI-H929, U266, MM1.S were labeled with CellTrace Violet according to the manufacturer’s protocol and 5×10^4^ cells were incubated with 10 μg/ml of dara for at least 1 hour at 37°C, 5% CO_2_ before addition of effector NK cells that had been preactivated with XmAb24306 (10µg/ml). Various E:T ratios (0:1, 0.2:1, 0.5:1, 1:1, 2:1, 5:1) were used for these assays and co-cultures were allowed to proceed for a total of 5 h. The viability of MM cells prior to the initiation of the ADCC assay was >90%. Cells were then harvested and stained with extracellular surface antibodies followed by Annexin V and 7AAD staining to label dead target cells. Amount of NK cell ADCC was calculated using the formula used to calculate cytotoxicity above. % NK cell fratricide= (% NK cell death in presence of dara with or without XmAb24306) – (% NK cell death in the absence of dara with or without XmAb24306). For evaluating the polyfunctional NK cell cytokine responses, brefeldin A and monensin (to inhibit intracellular protein transport) were added during the 5 h incubation and subsequently, extracellular and intracellular staining were completed as described below. Experiments measuring ADCC were conducted with n=3-4 individual donors as specified and are pooled data from 2-3 independent experiments.

### 2.6 Multiparameter flow cytometry

Cell counts for prepared single-cell suspensions of NK cells were determined using a Vi-CELL XR (Beckman Coulter). Briefly, 1×10^5^ enriched blood NK cells from single-cell suspensions were incubated with a mixture of fluorescence-conjugated anti-human antibodies for 30 min at 4°C. Stained cells were then washed twice, and following extracellular surface staining, where appropriate, the Foxp3 Fixation/Permeabilization Concentrate and Diluent Kit (Thermo Fisher Scientific) was used for intracellular staining. Samples were subsequently acquired with an LSR Fortessa flow cytometer (BD) using FACSDiva software (BD) or Cytek Aurora flow cytometer (Cytek Biosciences) using SpectroFlo Software v2.2.0.2. To evaluate NK cell degranulation and cytokine production, NK cells were cocultured with the appropriate target cells for 5 h in the presence of anti-CD107a and 1X protein transport inhibitor cocktail (ThermoFisher Scientific). This was followed by surface and intracellular staining for cytokine production as described above. Analysis was completed using FlowJo software version 10.2 (TreeStar) or cloud-based OMIQ (Dotmatics). A comprehensive list of all antibodies used can be found in **Supplementary Table 1**.

### 2.7 Tumor spheroid generation

To generate spheroids using HCT-116 colorectal tumor cells, 5×10^3^ CellTrace Far Red (2.5 µM) (ThermoFisher Scientific) labeled cells were plated and centrifuged (290 g for 3 min) on Day 0 in 96 well Nunclon Sphera plates (ThermoFisher Scientific). Nunclon Sphera plates were subsequently centrifuged (100 g, 3 min) and cultured at 37°C, 5% CO_2_. Spheroids were monitored for development over 5 days. On Day 3, 1x10^5^ Carboxyfluorescein succinimidyl ester (CFSE) (2.5 μM) (ThermoFisher Scientific) labeled NK cells that had been stimulated overnight with or without XmAb24306 (10µg/ml) were added to spheroids and cocultures were then subjected to time lapse confocal imaging as described below. Three technical replicates were established for each donor evaluated.

### 2.8 Time lapse microscopy of NK cell induced tumor spheroid killing

Spheroids were imaged every 4 hours for 5 days at 37°C and 5% CO_2_. Z-stacks were acquired in phase, 488 and 647 channels at 10 μm steps (140 μm total) with a 10× Plan Fluor objective (NA: 0.3, Nikon). Imaging was performed on a Nikon Ti-E perfect focus inverted microscope equipped with a spinning disk confocal CSU-X1 (Andor, Oxford Instruments) motorized X, Y stage (Nikon), environmental chamber (OkoLab) and Prime 95B sCMOS camera (Teledyne Photometrics), all controlled by NIS-Elements software (Nikon). Z-stacks were processed into maximum intensity projections, spheroids were traced in the phase image and area was measured at every time point, then normalized to the initial measurement at t=0. Total integrated intensity of the CellTrace Far Red fluorescence was measured within spheroid ROIs in the 647 channel and normalized to t=0 measurements. 6 donors with 3 wells each were assayed with or without XmAb24306 treated NK cells, or spheroids alone.

### 2.9 Statistical analysis

The results are presented as mean +/- standard deviation (s.d.) or with box plots, as appropriate. Data sets with sample size greater than or equal to 10 were evaluated for normality using Kolmogorov-Smirnov, Shapiro-Wilk and D’Agostino & Pearson normality tests. If there was insufficient evidence to reject normality, a one-way ANOVA with Tukey multiple comparisons test was applied. In the case of failed normality tests or small sample sizes, Wilcoxon-matched paired rank tests were used to evaluate differences between two paired groups, and the Friedman test with Dunn’s multiple comparisons to evaluate multiple matched group comparisons. For statistical evaluation, P<0.05 was considered significant. Not significant (NS)= P>0.05, *P<0.05, **P<0.005, ***P<0.0005 and ****P<0.0001. Corrections for multiple hypothesis testing were not performed for this exploratory analysis. Correlation values were calculated with Pearson tests. The number of donors used were N=6-11 per group and is indicated in each respective figure legend. Python was used for graphical visualization of data. All statistical analyses were performed using GraphPad Prism 9.4 (Dotmatics).

## 3 Results

### 3.1 XmAb24306 enhances direct cytotoxicity of primary human NK cells against several tumor target cell lines

**Figure 1:**
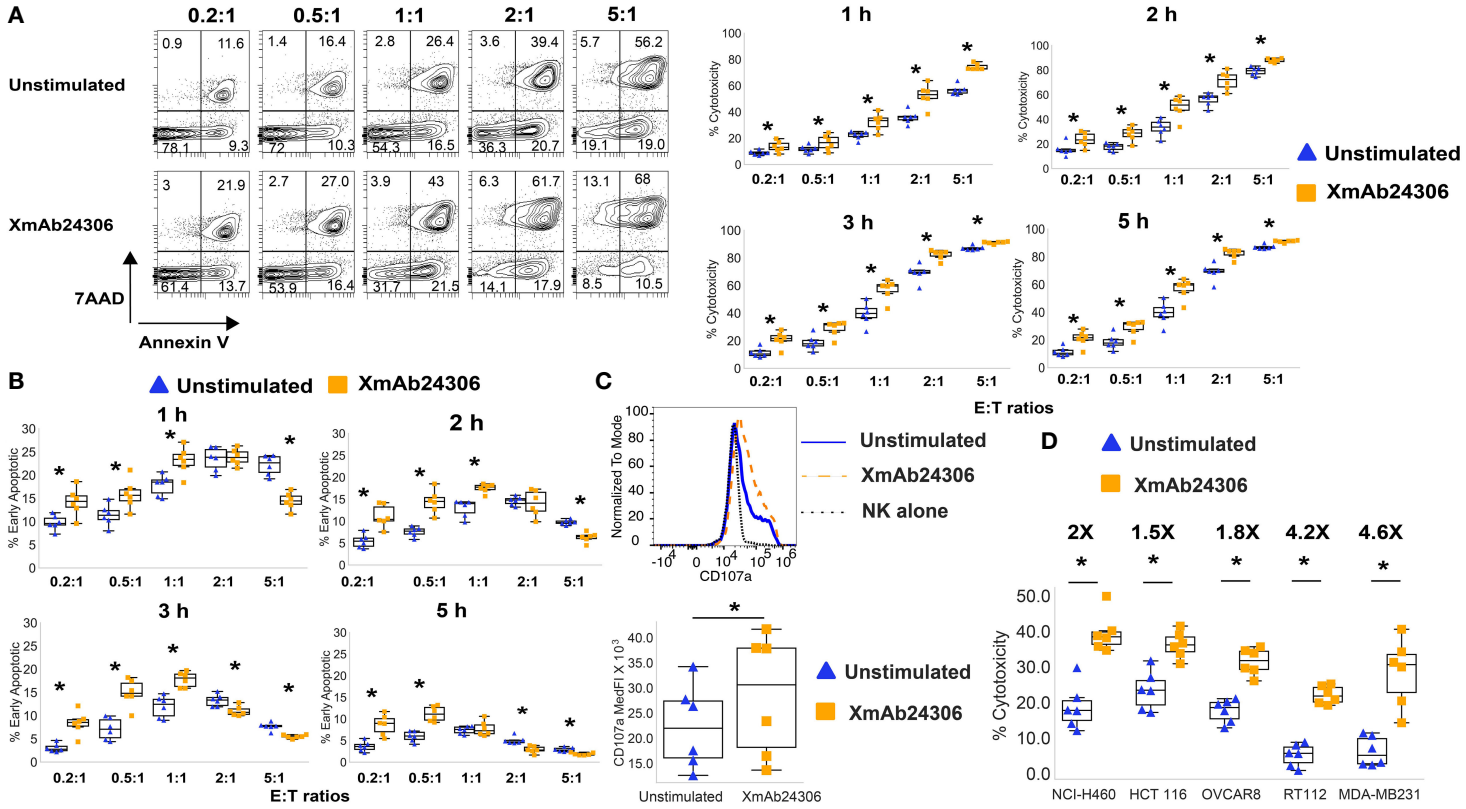
XmAb24306 enhances the capacity of NK cells to kill K562 and solid tumor target cell lines more rapidly. **(A)** Representative flow cytometry plots showing 7AAD and Annexin V dye uptake in CTV+ K562 targets after culture with NK cells treated or untreated with XmAb24306 (10µg/ml) from one donor (left) after 1 h of coculture with K562 targets. Quantification of NK cell mediated cytotoxicity (7AAD+ Annexin V+) against K562 targets at various E:T ratios and time points (right) (n=6). **(B)** The percent of Annexin V+ 7AAD- CTV+ K562 targets (apoptotic cells) after coculture with NK cells treated with or without XmAb24306 (n=6). **(C)** CD107a expression in NK cells treated with or without XmAb24306 and then co-cultured with K562 targets for 5 h (n=6). **(D)** The quantification of NK cell-mediated cytotoxicity (7AAD+ Annexin V+) against lung (NCI-H460), colon (HCT116), ovarian (OVCAR8), bladder (RT112) and breast (MDA-MB231) cancer cell lines after coculture with NK cells treated with or without XmAb24306 (E:T= 2:1) (n=6). Each dot represents a different donor, and for all plots, error bars indicate mean ± SD. K562 and solid tumor cell line killing assays were tested with at least 6 individual donors. Statistical analyses in **(A–D)** were performed using Wilcoxon matched pairs signed rank test. *P<0.05.

To determine whether XmAb24306 may enhance the killing efficiency of NK cells directly *ex vivo*, we enriched primary NK cells from the blood of healthy participants (**Supplementary Figure 1**) and incubated NK cells with or without XmAb24306 (10µg/ml) for 18–20 hrs. After incubation, live NK cells from untreated and XmAb24306 treated conditions were counted and equal numbers were then co-cultured with cell trace violet (CTV) labeled chronic myelogenous leukemia cell line, K562, at various effector to target (E:T) ratios. The efficiency with which NK cells killed K562 targets was measured at defined time points (**Figures 1A, B**). Treatment of NK cells with XmAb24306 led to more efficient target cell killing (**Figure 1A**) and more rapid induction of early apoptosis (**Figure 1B**) compared to the activity of the same NK cells that were not primed with XmAb24306. XmAb24306 significantly increased the efficiency of killing at all E:T ratios examined, including lower E:T ratios (0.2:1 and 0.5:1) that more likely model the *in vivo* situation. In these lower E:T ratio conditions, efficiency of NK cell killing was increased by about two-fold in the 5 h cytotoxicity assay (**Figure 1A**, right).

Granule exocytosis is the predominant mechanism used by NK cells to eliminate their targets (20). The cell surface expression of CD107a, a lysosomal protein, is considered a sensitive marker of NK cell degranulation and release of cytotoxic granules. To determine the degranulation capacity of XmAb24306 treated NK cells against K562 cells, we co-cultured NK cells that were treated with or without XmAb24306 with K562 target cells for 5 h and measured the expression of CD107a as a surrogate marker for NK cell degranulation by flow cytometry. Our data shows that XmAb24306 increases CD107a expression, suggesting enhanced degranulation capacity of NK cells within 5 h against K562 target cells (**Figure 1C**).

K562 tumor cells lack HLA-I expression, which predisposes them to be highly sensitive to NK cell mediated killing (43, 44). In contrast, NK cell killing of HLA-I expressing tumor cells is less efficient due to the presence of inhibitory signaling. To determine if XmAb24306 could also increase NK cell cytotoxicity against HLA-I expressing cell lines, we used various solid tumor cell lines derived from lung, colon, ovarian, bladder and breast cancers. We observed that while NK cell cytotoxicity against these targets was less than that observed for K562, XmAb24306 consistently and significantly enhanced the cytotoxic capacity of NK cells against these various tumor cell lines (E:T=2:1) (**Figure 1D**) (45, 46). These results suggest that XmAb24306 can enhance NK cell killing against a broad range of tumor cell lines *in vitro*.

### 3.2 XmAb24306 enhances NK cell killing of 3D colorectal tumor spheroids

**Figure 2:**
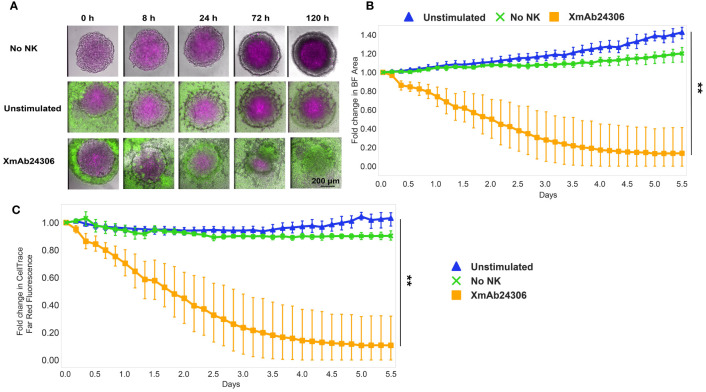
XmAb24306 enhances NK cell killing of 3D colorectal spheroids. **(A)** Representative spinning disk confocal timelapse images of HCT116 colorectal spheroids (labeled with CellTrace Far Red- Magenta) cultured with NK cells (CFSE- green) that were treated overnight with or without XmAb24306 (10µg/ml). Images are overlays of brightfield (BF), 488 and 647 channels at indicated time points. **(B)** Quantitative image analysis of the spheroid area or **(C)** CellTrace Far Red dye intensity within the spheroid acquired at the indicated times and normalized to initial measurements. Means ± SD are depicted (n = 6). NK cell killing of spheroids for each donor were run in triplicate and the mean value for brightfield area or CellTrace Far Red intensity was reported for each donor. Data is representative of two independent experiments. Statistical analyses in **(B, C)** were performed using the Friedman test with Dunn’s multiple comparisons test. **P<0.005.

In addition to enhancing killing of tumor cell lines, we wanted to know whether XmAb24306 would enhance killing of three-dimensional spheroids as well. We evaluated the effects of NK cells (CFSE-labeled) on the integrity of colorectal (HCT116) tumor spheroids fluorescently labeled with CellTrace Far Red dye, using bright-field and fluorescence confocal time lapse microscopy (**Figure 2A**; **Movie S1**). HCT116 are known to have high levels of HLA-I and NKG2DL (47). Within 24 h after NK cell addition, XmAb24306-treated NK cells began to infiltrate spheroids and intersperse with the HCT116 cells. The integrity and delineation of the tight tumor spheroid border significantly decreased after coculture with XmAb24306 treated NK cells (**Figure 2A**; **Movie S3**) in contrast to the untreated NK cells (**Figure 2A**; **Movie S2**). This disruption of the spheroid border coincided with a decrease in the relative spheroid area (**Figure 2B**) and CellTrace (HCT116 -label) intensity (**Figure 2C**) over the time course, indicating increased cytolytic activity of the XmAb24306-treated NK cells. Taken together, these data suggest that XmAb24306 enhances the capacity of NK cells to disintegrate and kill 3D colorectal tumor spheroids *in vitro*.

### 3.3 XmAb24306 enhances ADCC-mediated and direct killing capacity of NK cells against multiple myeloma cell lines *in vitro*

**Figure 3:**
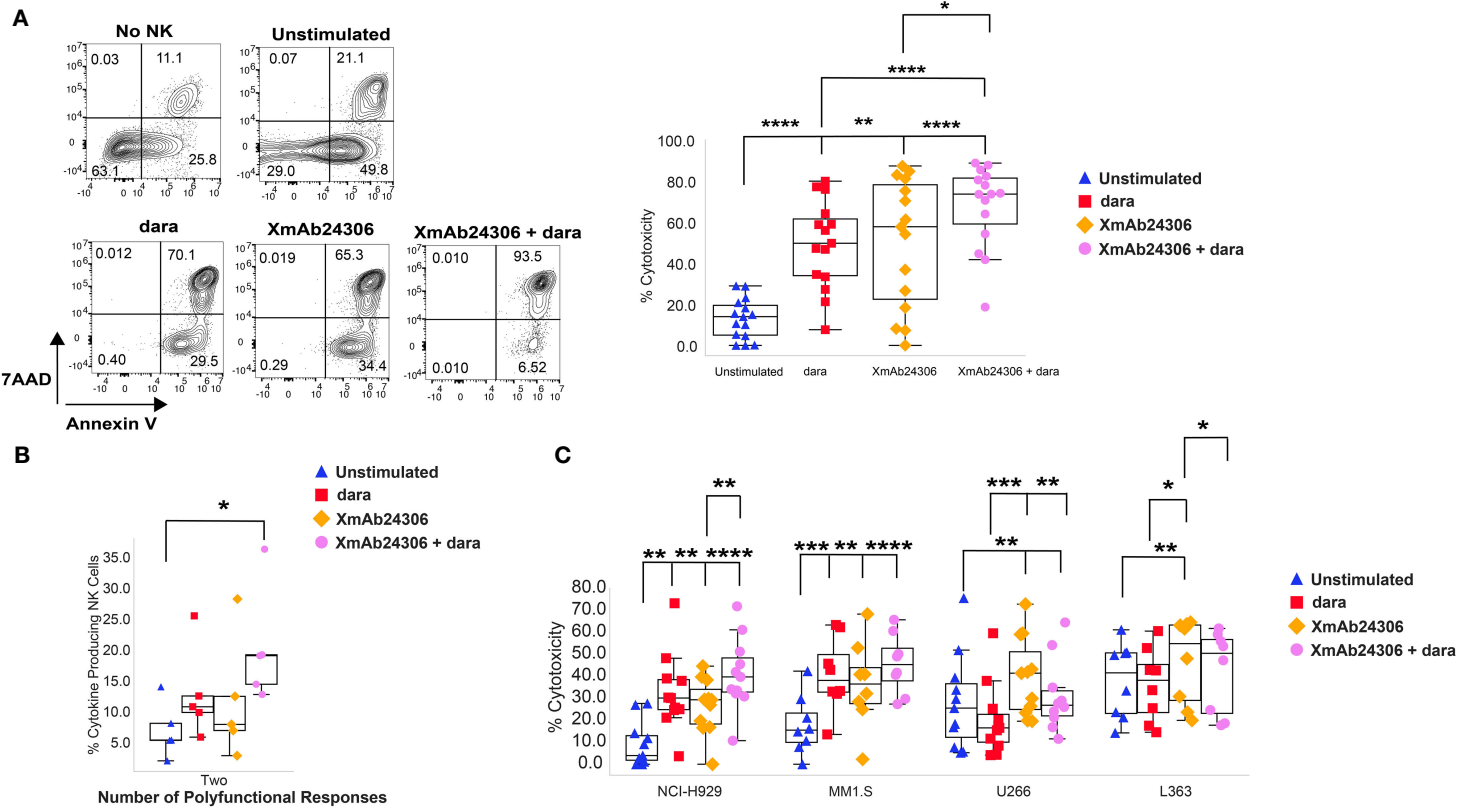
XmAb24306 enhances the daratumumab-mediated NK cell ADCC against MM target cell lines. **(A)** Representative flow cytometry plots for a single donor showing the 7AAD and Annexin V dye uptake in CTV+ RPMI 8226 targets after coculture with NK cells stimulated with or without XmAb24306 (10µg/ml) (E:T = 2:1) in the presence or absence of dara (left). Quantification of NK cell mediated killing (7AAD+ Annexin V+) in each of the indicated conditions (right) (n=15). **(B)** Polyfunctional NK cell cytokine responses representing the fraction of NK cells producing any combination of one, two, three or four of IFN-γ, TNF-α, CD107a and MIP1-β (n=5) **(C)** NK cell-mediated killing (7AAD+ Annexin V+) of NCI-H929, MM1.S and U266 MM cells in the presence of XmAb24306, dara or XmAb24306 + dara (n=11). Box plots represent mean with interquartile range, and for all plots, each dot represents data from a different donor. All experiments were conducted with n=3-4 individual donors and are pooled data from 2-3 independent experiments. Statistical analyses were performed using one way ANOVA with Tukey multiple comparisons tests. P>0.05, *P<0.05, **P<0.005, ***P<0.0005 and ****P<0.0001.

To understand how the ADCC function of NK cells would be influenced by XmAb24306, we measured dara-mediated cytotoxicity against RPMI 8226, a MM cell line with high cell surface CD38 expression (48). Target RPMI 8226 were labeled with CTV and co-cultured with either untreated NK cells or those pretreated with XmAb24306 (E:T= 2:1). XmAb24306 and dara alone can each enhance NK cell killing of RPMI 8226 cells, and the combination of dara and XmAb24306 shows an improvement in cell killing over the single agents (**Figure 3A**). These data show that XmAb24306 can enhance the direct killing capacity of NK cells against the RPMI 8226 MM cell line even in the absence of dara, and that the combination treatment of XmAb24306 and dara *in vitro* show an additive effect leading to a significantly enhanced ADCC effect compared to dara alone (**Figure 3A**).

In addition to their cytotoxic functionality, NK cells also produce effector cytokines upon activation (21–23). In this regard, we compared cytokine production (IFN-γ, TNF-α and MIP1-β) and the cytotoxic degranulation activity (CD107a) of NK cells in response to treatment with XmAb24306, dara and the combination of XmAb24306 and dara during cocultures with RPMI 8226 (**Figure 3B**). The fraction of polyfunctional NK cells producing any combination of two effector molecules was significantly higher in the presence of XmAb24306 and dara (**Figure 3B**).

As RPMI 8226 may not represent the heterogeneity of target expression and inhibitory molecules in MM, we asked whether XmAb24306 would enhance NK cell ADCC in response to other MM cell lines. We evaluated NK cell ADCC in response to NCI-H929 (CD38^hi^), MM1.S (CD38^hi^), U266 (CD38^lo^) and L363 (CD38^lo^) (E:T=2:1) which also express varying levels of NKG2DL, HLA-I and HLA-E (**Supplementary Data 2**) (49–54). XmAb24306 alone significantly enhanced the direct killing capacity and ADCC against all MM cell lines. However, the combination with dara did not significantly increase ADCC above the dara alone condition for the other CD38^hi^ expressing cell lines (**Figure 3C**, NCI-H929, MM1.S). Of note, dara led to a reduction in NK cell mediated killing of CD38^lo^ U266 and L363 cells, an expected result given the low expression of CD38 on these targets and potentially enhanced NK cell death due to dara-mediated fratricide of the CD38+ NK cells. Despite the lower CD38 expression on U266 and L363 cells (**Supplementary Data 2**) (49–54), XmAb24306 alone enhanced NK cell killing of these target cell lines in the absence of dara (**Figure 3C**). Taken together, this data shows that the combination of XmAb24306 with dara increased ADCC on CD38^hi^ MM cell lines and that XmAb24306 alone was able to enhance NK cell killing against CD38^lo^ MM cell lines.

### 3.4 Increased fratricide in the presence of XmAb24306 and daratumumab does not impact NK cell mediated ADCC

**Figure 4:**
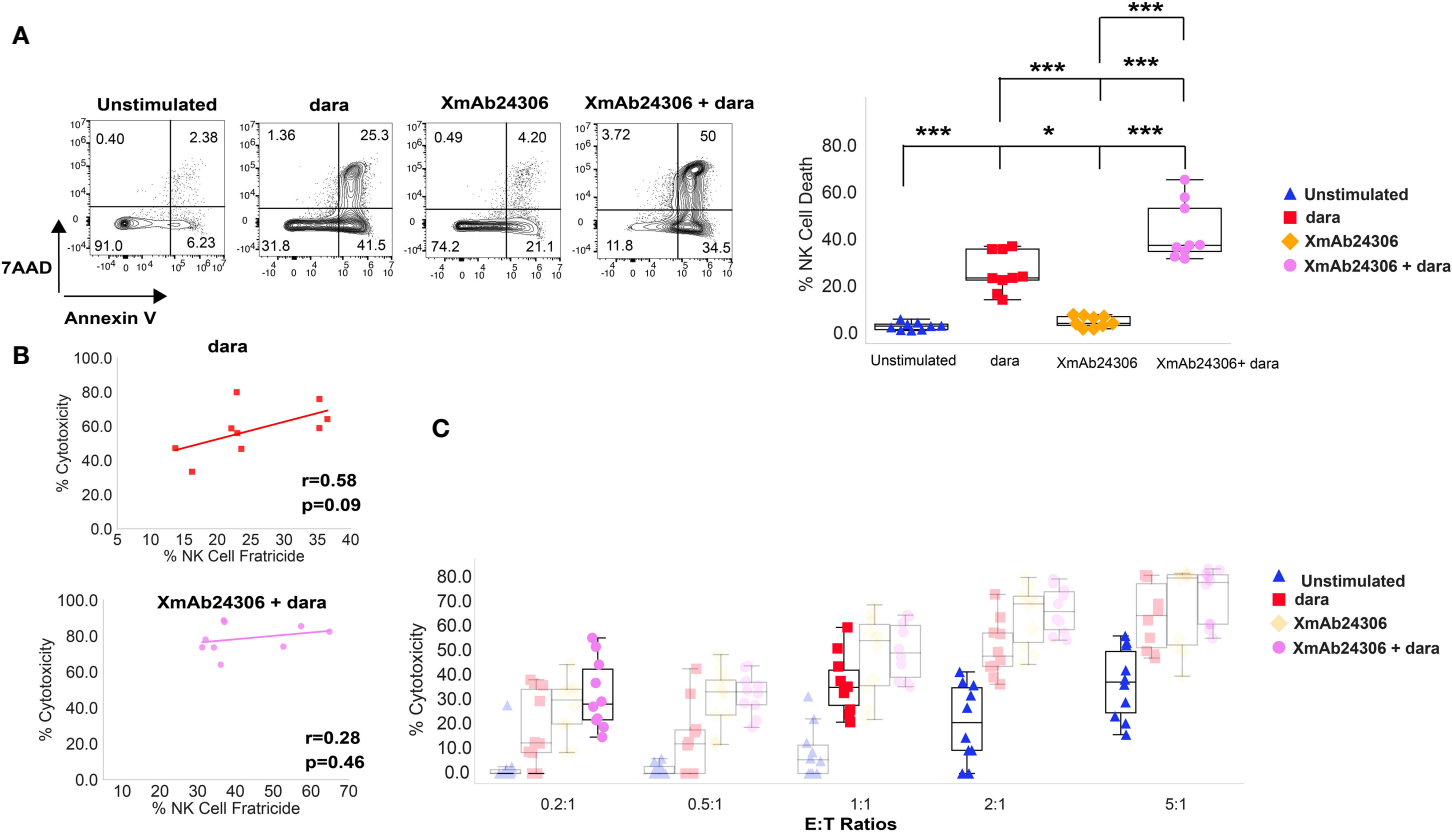
Enhanced NK cell fratricide in the presence of XmAb24306 and daratumumab does not impair NK cell ADCC potential *in vitro*. **(A)** Representative flow cytometry plots for a single donor showing the 7AAD and Annexin V dye uptake in peripheral blood NK cells in the presence or absence of dara, XmAb24306 (10µg/ml) and the combination of both (left). Quantification of NK cell death in each of the indicated conditions (right) (E:T = 2:1) (n=9). **(B)** Correlation analysis between % cytotoxicity and % NK cell fratricide for NK cells treated with dara or dara + XmAb24306 in co-culture experiments with RPMI 8226 MM cells. Pearson correlation coefficient and two-tailed p-values shown (n=9). **(C)** Box plots showing % RPMI 8226 cell killing (7AAD+ Annexin V+) at various E:T ratios over a 5 h assay (n=10). Box plots represent data with interquartile range. All experiments were conducted with n=3-4 individual donors and are pooled data from 2-3 independent experiments. Statistical analyses were performed using one way ANOVA with Tukey multiple comparisons tests. Each dot represents a different donor (n = 9–10). P>0.05, *P<0.05 and ***P<0.0005.

A subset of NK cells express CD38 (37). Dara has been shown to mediate NK cell fratricide by binding to CD38 expressed on NK cells (37). We therefore asked whether the combination of XmAb24306 and dara would augment NK cell fratricide. Target RPMI 8226 were labeled with CTV and co-cultured with either untreated NK cells or those pretreated with XmAb24306 (E:T=2:1) in the presence or absence of dara and NK cell death was evaluated within 5 h. Our results show that in the absence of dara, XmAb24306 had minimal effects on NK cell death (**Figure 4A**). However, when combined with dara, XmAb24306 significantly increased the degree of NK cell death (fratricide) compared to dara alone (**Figure 4A**).

To determine whether the enhanced fratricide in the presence of XmAb24306 and dara decreased ADCC mediated killing by NK cells, we performed correlative analyses between % cytotoxicity mediated by ADCC and % NK cell fratricide. We did not observe any negative correlation between higher rates of NK cell fratricide and NK cell ADCC either in the presence of dara alone or the combination of XmAb24306 and dara (**Figure 4B**). Additionally, because NK cell fratricide may reduce the effective E:T ratio, we tested whether XmAb24306 treatment can still induce robust tumor killing even at lower E:T ratios *in vitro*. To this end, we co cultured XmAb24306 treated and untreated NK cells with CTV labeled RPMI 8226 at various E:T ratios for 5 h and measured NK cell mediated cytotoxicity in the presence or absence of dara. At the lowest E:T ratio of 0.2:1, addition of dara drives minimal MM cell killing (5–10%). However, the combination of XmAb24306 and dara leads to 5-fold increased ADCC compared with dara alone at this E:T ratio. Equally important, the combination treatment at 0.2:1 E:T ratio induces similar levels of ADCC compared with single agent dara when tested at 5 times higher E:T ratio (1:1) (**Figure 4C**) (purple dots Vs. red diamonds). Similarly, the combination of XmAb24306 and dara at E:T ratio of 2:1 also yielded similar ADCC when compared with dara alone at more than 2 times the number of effectors (E:T ratio of 5:1) (purple dots Vs. blue triangles). Taken together, these data show that XmAb24306 can enhance the ability of NK cells to efficiently and robustly eliminate MM cell lines even under conditions of low E:T ratio which may happen due to fratricide.

## 4 Discussion

IL-15 can enhance NK cell functionality; thus, it has been of interest to develop strategies to enhance NK cell mediated anti-tumor immunity using this cytokine (11, 12, 55, 56). XmAb24306 is an engineered IL-15/IL-15Rα fusion protein that aims to address the clinical limitations of using recombinant IL-15 as an anticancer therapy by decreasing binding affinity for the receptor and increasing half-life of the molecule (14, 15). We sought to determine how XmAb24306 would modulate human NK cell functionality against tumor cell lines *in vitro*. Our results show that XmAb24306 enhances the capacity of primary human NK cells to rapidly and more efficiently kill various tumor cell lines and 3D spheroid targets.

Our data suggest that XmAb24306 enhances the kinetics of primary human NK cell-mediated tumor cell line killing *in vitro*. In co-culture experiments with K562 cells, XmAb24306-stimulated NK cells demonstrated enhanced killing capacity by 1.5–2-fold at every time point evaluated, especially at lower E:T ratios which may be more clinically relevant. These data suggest that XmAb24306 may enhance the ability of NK cells to rapidly kill targets and enhance their serial killing properties, potentially enabling NK cells to eliminate multiple targets in a shorter period of time. This observation may have important implications particularly in physiological conditions where E:T ratio in the TME may be very low (18, 57). The ability of XmAb24306 to boost NK cell serial killing even at lower E:T ratios could lead to better control of tumor growth. As K562 targets are more highly sensitive to NK cell lysis given the lack of strong inhibitory signals from the absence of HLA-I, we also examined if XmAb24306 would enhance NK cell killing against a variety of HLA-I expressing solid tumor cell lines. XmAb24306 enhanced NK-cell mediated killing of various cell lines derived from lung, colon, ovarian, bladder and breast cancer, as well as in colorectal tumor 3D spheroids. Our results show that XmAb24306 enhanced the ability of NK cells to reduce spheroid size and integrity as well as enhance their capacity to intersperse the spheroids, highlighting the potential of XmAb24306 to enhance NK cell function during cancer immunotherapy.

In addition to directly killing tumor cells driven by NK cell activating receptors, ADCC mediated by CD16 engagement on NK cells is a key mechanism of action of NK cell-mediated tumor cell killing, especially for tumor targeting antibodies. Dara, a CD38 targeting antibody induces MM cell death through various modes of action including ADCC mediated by NK cells, antibody-dependent cellular phagocytosis (ADCP), complement dependent cytotoxicity (CDC), immunomodulatory effects, and direct induction of apoptosis (58). Our data shows that the anti-cancer efficacy of dara-mediated ADCC against MM cell line targets was effectively enhanced when combined with XmAb24306. While dara has been shown to have strong efficacy and tolerability for the treatment of MM, there are some patients who do not respond to dara and show signs of progressive disease (59–63). The downregulation of CD38 expression on MM cells may be one mechanism to explain the resistance to dara in some patients (39). We show that XmAb24306 is able to enhance cytotoxicity against multiple MM cell lines including U266 and L363 MM cells that express lower levels of CD38 than RPMI 8226 cells. The enhanced susceptibility of CD38^lo^ MM cell lines suggests that combining XmAb24306 with dara may be beneficial if patients have either low expression of CD38 on MM cells as a primary resistance or develop resistance to dara through loss of CD38 expression on MM cells.

Dara can also mediate NK cell fratricide by binding to CD38 which is also expressed on NK cells (40). Fratricide significantly reduces the number of NK cells in MM patients following dara treatment (40); however, NK cell numbers usually recover by 50% within 3 months after the last administered dara dose and completely recover within 6 months as reported in the GEN501 and SIRIUS clinical trials (40). Importantly, our *in vitro* studies show that enhanced fratricide in the presence of XmAb24306 and dara did not negatively impact MM cell killing by ADCC. Furthermore, our data suggests that even under conditions of low NK cell to tumor ratio, XmAb24306 enhances the ability of NK cells to kill MM cells to levels comparable with 5 times higher E:T ratios. This is likely driven by enhanced serial killing ability of NK cells on a per-cell basis that can compensate for lower overall NK cells numbers. Altogether, our data suggests that NK cells that survive fratricide seem to be sufficient and efficient at mediating MM cell killing *in vitro*. It is also possible that XmAb24306 may enhance NK cell expansion *in vivo* to compensate for fratricide and ultimately lead to faster recovery of NK cells after dara treatment in patients. Currently, a Phase 1b study investigating the combination of XmAb24306 and dara in patients with relapsed/refractory MM is ongoing.

A limitation of this study is the absence of experiments showing the efficacy of XmAb24306 in murine tumor models. XmAb24306 is not cross reactive in mice, and surrogate molecules do not fully recapitulate the activity of XmAb24306 as observed on human cells. Therefore, using mouse tumor models would not recapitulate the safety or efficacy that would be expected in humans. Another limitation is the absence of long-term effects of XmAb24306 on NK cells. IL-15 can promote maturation and differentiation of immature NK cells *in vivo*, therefore, long-term *in vitro* cultures with mainly mature NK cells isolated from PBMCs of healthy donors would not faithfully recapitulate the long-term effect of XmAb24306 in cancer patients. For these reasons, the safety and long-term tolerability of XmAb24306 is currently being evaluated in Phase 1 clinical development in cancer patients.

## Data availability statement

The original contributions presented in the study are included in the article/**Supplementary Material**. Further inquiries can be directed to the corresponding author.

## Ethics statement

The studies involving humans were approved by Samples for Science (S4S) Procedure Sponsored by Genentech. CEHS-CP 307.2 WCG IRB Protocol #20080040. The studies were conducted in accordance with the local legislation and institutional requirements. The participants provided their written informed consent to participate in this study.

## Author contributions

HS: Conceptualization, Data curation, Formal Analysis, Investigation, Methodology, Validation, Visualization, Writing – original draft. PD: Conceptualization, Investigation, Methodology, Visualization, Writing – original draft. SG: Investigation, Methodology, Visualization, Writing – original draft, Formal Analysis. PH: Visualization, Resources, Writing – review & editing. SS: Resources, Writing – review & editing, Conceptualization, Supervision.

## Supplementary material

Movie S1-3 XmAb24306 enhances NK cell killing of 3D colorectal spheroids, related to . Representative movies of HCT116 spheroid alone (CellTrace Far Red-Magenta, **Movie S1**), or spheroids cultured with NK cells (CFSE-green) that were treated overnight -/+ XmAb24306 (-XmAb24306, **Movie S2**) (+XmAb24306, **Movie S3**). Z-stack images were acquired every 4 hours for approximately 120 h and processed into focused projections for visualization. The video plays at 3.33 frames per second.
