# Supplementary figures and images for: Efbalropendekin Alfa enhances human natural killer cell cytotoxicity against tumor cell lines in vitro
Source: Front Immunol. 2024 Mar 7;15:1341804. doi: 10.3389/fimmu.2024.1341804 (PMC10954783; doi:10.3389/fimmu.2024.1341804)

# Pre-enrichment

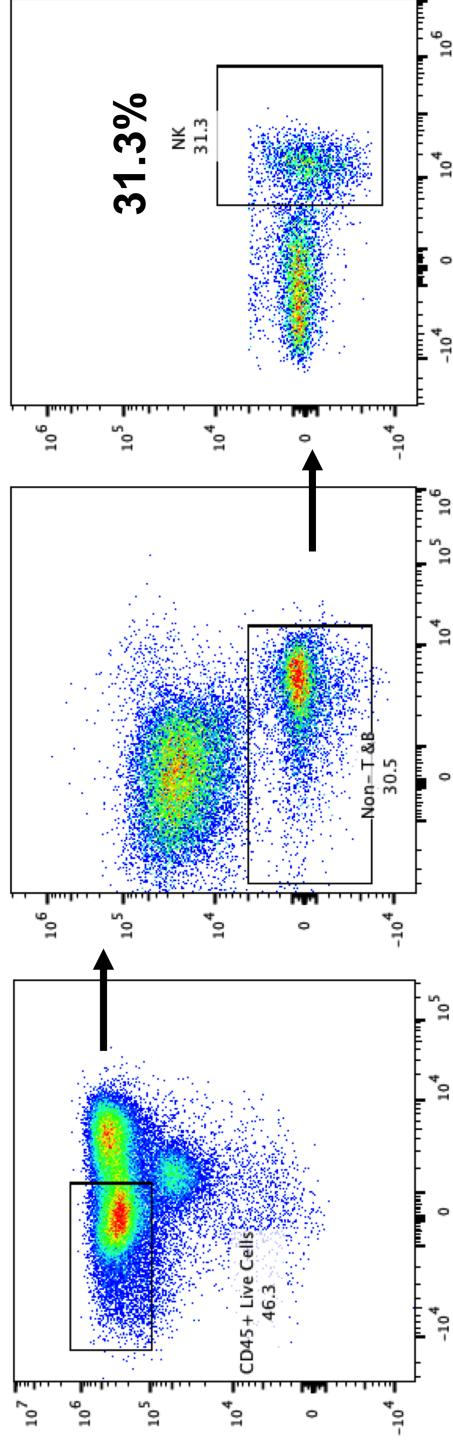

# Post-enrichment

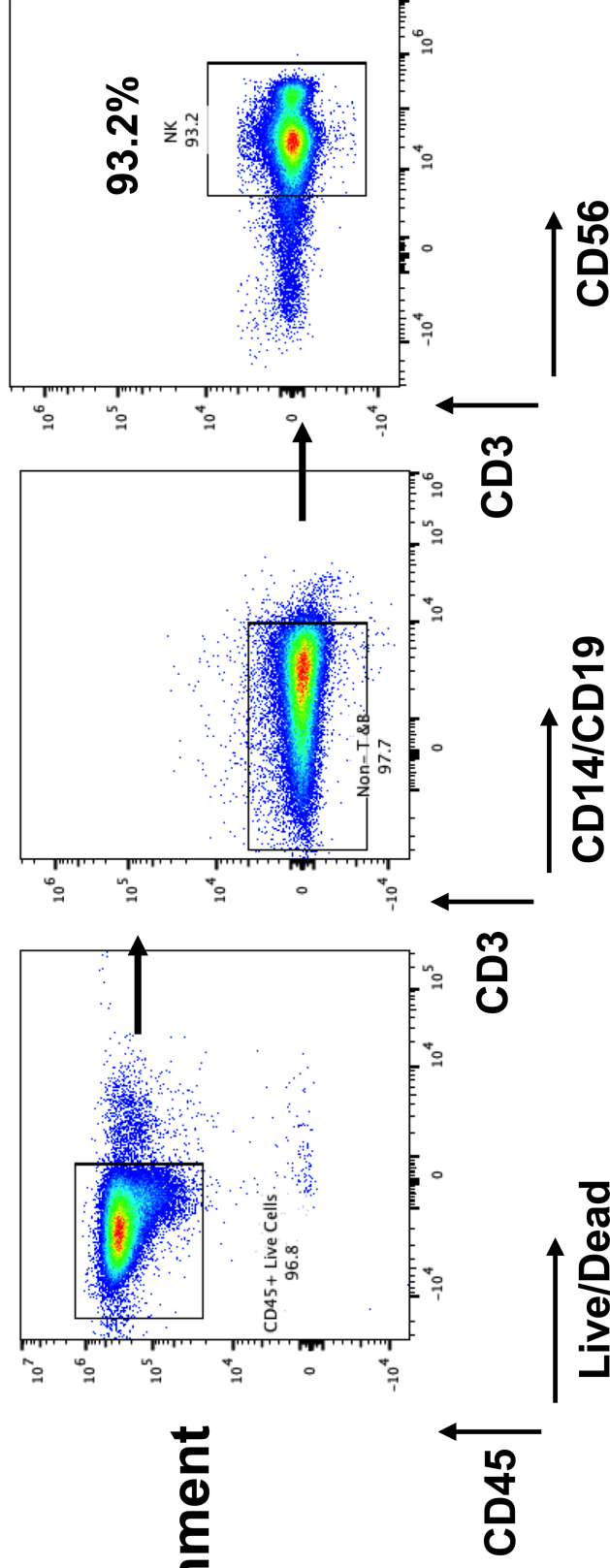

Supplement: Supplementary file 1 [file DataSheet_1.pdf]

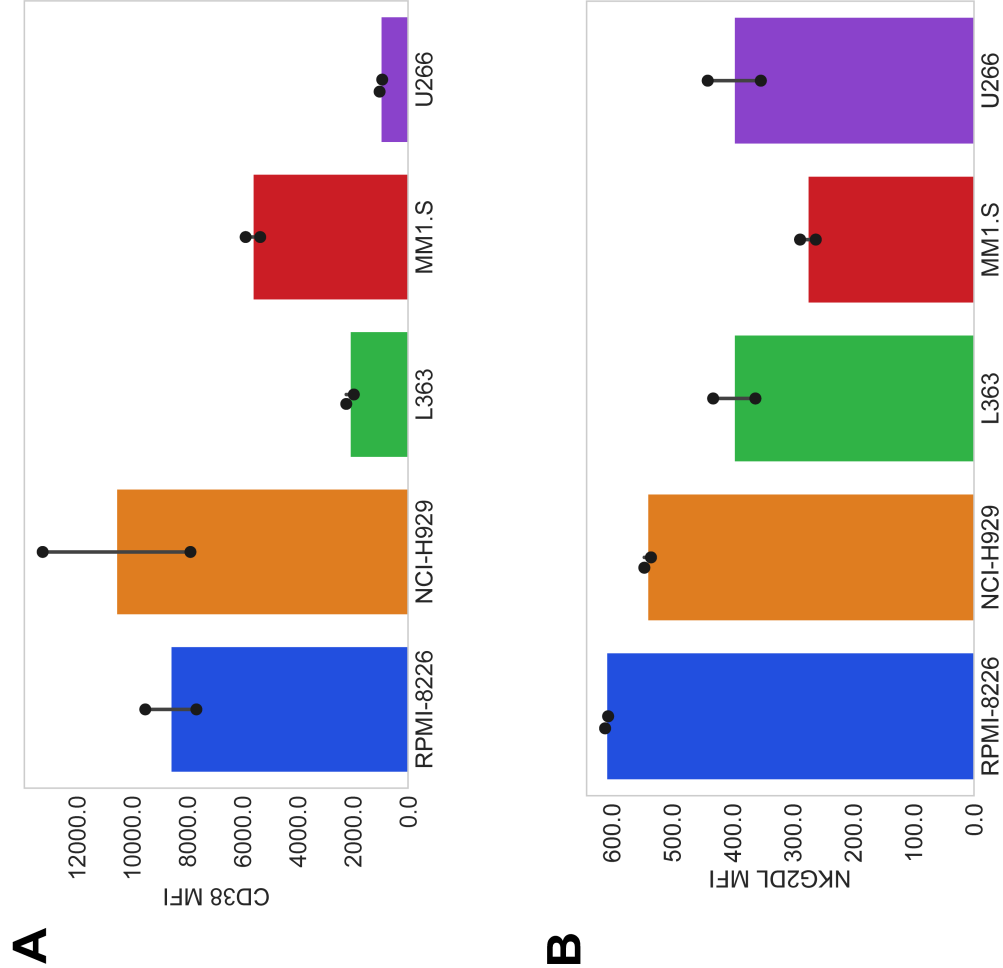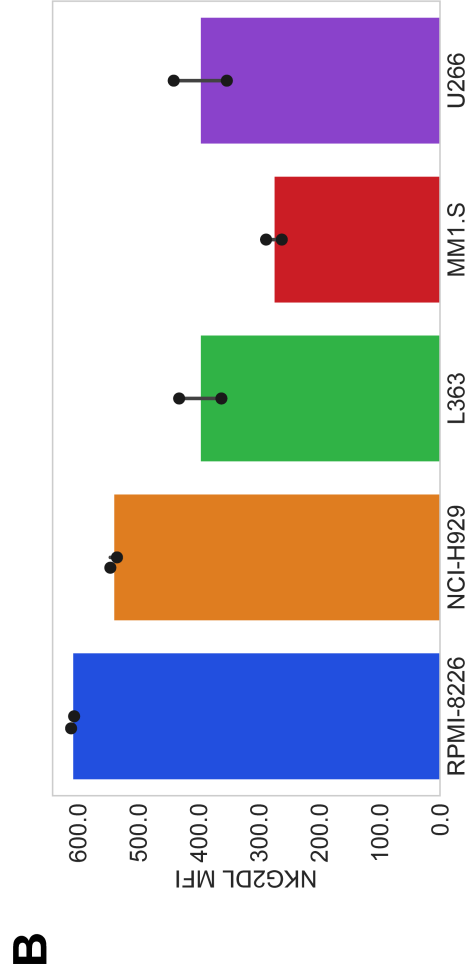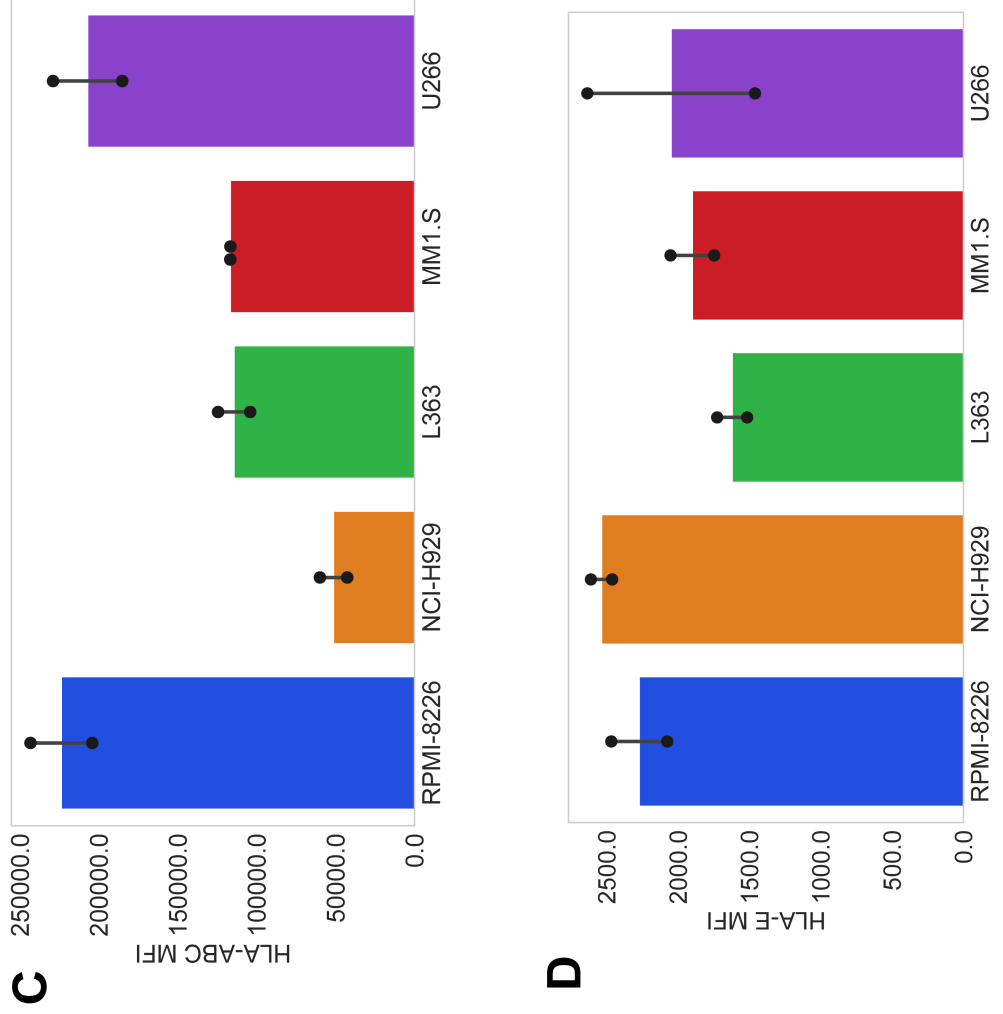

Supplement: Supplementary file 2 [file DataSheet_2.pdf]
