# Supplementary material for: Efbalropendekin Alfa enhances human natural killer cell cytotoxicity against tumor cell lines in vitro
Source: Front Immunol. 2024 Mar 7;15:1341804. doi: 10.3389/fimmu.2024.1341804 (PMC10954783; doi:10.3389/fimmu.2024.1341804)
Supplement: Supplementary file 6 [file Table_1.docx]

| **Table 1** |  |  |  |  |  |
| --- | --- | --- | --- | --- | --- |
|  | Marker | Fluorophore | Clone | Manufacturer | Catalog # |
| 1 | Live Dead | Live dead blue | n/a | ThermoFischer | L23105 |
| 2 | CD3 | BUV395 | SP34-2 | BD | 564117 |
| 3 | CD14 | AF532 | 61D3 | ThermoFischer | 58-0149-41 |
| 4 | CD19 | AF532 | HIB-19 | ThermoFischer | 58-0199-42 |
| 5 | CD56 | BUV737 | NCAM16.2 | BD | 612766 |
| 6 | CD16 | BUV496 | B73.1 | BD | 741207 |
| 7 | CD38 | FITC | Cytognos Multiepitope | Cyntognos | CYT-38F2-A |
| 8 | CD45 | PE-Cy5 | HI30 | Biolegend | 304010 |
| 9 | Granzyme B (GZB) | BV510 | GB11 | BD | 563388 |
| 10 | CD107a | EF660 | eBIOH4A3 | ThermoFischer | 50-1079-42 |
| 11 | IFN-𝛄 | PECF594 | B27 | BD | 562392 |
| 12 | TNF-⍺ | PercPcy5.5 | MAb11 | BD | 560679 |
| 13 | MIP-1β | PE-Cy7 | D21-1351 | BD | 560709 |
| 14 | GM-CSF | PE | BVD2-21C11 | BD | 561165 |
| 15 | Annexin V | PE | n/a | BD | 556421 |
| 16 | 7AAD | 7AAD | n/a | BD | 559925 |
| 17 | hULBP2/5/6 | PE | n/a | R&D systems | FAB1298P |
| 18 | hULBP3 | PE | n/a | R&D systems | FAB1517P |
| 19 | hULBP1 | PE | n/a | R&D systems | FAB1380P |
| 20 | MICA/B | PE | n/a | R&D systems | FAB13001P |
| 21 | HLA-E | PE-Cy7 | 3D12 | Biolegend | 342608 |
